# Backscattering Echo Intensity Characteristics of Laser in Soil Explosion Dust

**DOI:** 10.3390/s23125638

**Published:** 2023-06-16

**Authors:** Lijuan Gao, Fue-Sang Lien, Huimin Chen, Guang Chen, Shangxian Yang, Jiahao Deng

**Affiliations:** 1 Science and Technology on Electromechanical Dynamic Control Laboratory, Beijing Institute of Technology, Beijing 100081, China; gaolijuansd@bit.edu.cn (L.G.);; 2 Department of Mechanical and Mechatronics Engineering, University of Waterloo, Waterloo, ON N2L 3G1, Canada; fue-sang.lien@uwaterloo.ca; 3 Key Laboratory of Traffic Safety on Track, Central South University, Changsha 410083, China; 4 Beijing Bo Tsing Technology Co., Ltd., Beijing 100176, China

**Keywords:** backscattering echo intensity, laser, soil explosion dust, soil moisture content, mean gray value

## Abstract

Soil dust generated by explosions can lead to the absorption and scattering of lasers, resulting in low detection and recognition accuracy for laser-based devices. Field tests to assess laser transmission characteristics in soil explosion dust are dangerous and involve uncontrollable environmental conditions. Instead, we propose using high-speed cameras and an indoor explosion chamber to assess the backscattering echo intensity characteristics of lasers in dust generated by small-scale explosive blasts in soil. We analyzed the influence of the mass of the explosive, depth of burial, and soil moisture content on crater features and temporal and spatial distributions of soil explosion dust. We also measured the backscattering echo intensity of a 905 nm laser at different heights. The results showed that the concentration of soil explosion dust was highest in the first 500 ms. The minimum normalized peak echo voltage ranged from 0.318 to 0.658. The backscattering echo intensity of the laser was found to be strongly correlated with the mean gray value of the monochrome image of soil explosion dust. This study provides experimental data and a theoretical basis for the accurate detection and recognition of lasers in soil explosion dust environments.

## 1. Introduction

Lasers are useful for high-precision, long-distance measurements; they can accurately detect and recognize targets and have strong anti-electromagnetic interference ability [1,2,3]. In a military context, the role of lasers in fuzes and lidars is increasingly important in the land battlefield environment. The laser fuze especially is widely used in ammunition systems, such as anti-tank missiles, bombs, and projectiles [4,5,6]. However, laser beams can easily be reflected or scattered. On a battlefield, aerosol particles such as smoke [7,8], fog [9,10,11], and dust [12,13,14,15,16] are common and may interfere with the laser, reducing its detection and recognition performance. In particular, soil dust [17] kicked up by vehicles or scattered by the explosion of shallowly buried ammunition can absorb and scatter laser radiation, potentially leading to lidar detection failure or early detonation of a laser fuze. However, research on backscattering characteristics of laser beams propagating through soil explosion dust is still in the early stage.

To understand the temporal and spatial distribution of dust from the detonation of explosives buried in soil, some researchers have conducted field tests to study the craters formed by the detonation of explosives buried in soil, and have measured the particle size distribution and concentration of soil explosion dust at a specific position [18,19,20,21]. Because of the limitations of existing equipment, dust particles with diameters above 150 μm cannot be monitored; the measurement accuracy is not high; and conditions in the field, such as temperature, wind speed, and humidity, are uncontrollable. Moreover, such testing is dangerous, costly, and has poor repeatability. Another historical approach involves studying cratering and soil ejection in the early stages of explosions based on numerical simulation methods such as smoothed particle hydrodynamics (SPH), the Euler method, the Lagrange method, or other algorithms [22,23,24]. Studies taking this approach have paid little attention to the temporal and spatial distribution characteristics of soil explosion dust in the diffusion and settling phases. To examine the backscattering echo intensity characteristics of laser in a dust concentration field at different times and positions, most researchers have adopted numerical methods based on the optical geometric scattering, Mie scattering, Monte Carlo method, or T-matrix method and limited to a single and uniform dust concentration field [25,26,27,28]. The validity of such simulations is difficult to verify. Experimental research related to the real-time monitoring of the backscattering echo intensity characteristics of laser in soil explosion dust remains scarce.

To study the temporal and spatial distribution of soil explosion dust and measure the backscattering echo intensity characteristics of lasers in soil explosion dust, in the present study, we use high-speed cameras to assess the backscattering echo intensity characteristics of lasers in dust generated by the small-scale explosives blasted in soil in an explosion chamber. This method could measure the sizes of craters and capture the temporal and spatial distribution images of the formation, diffusion, and setting process of soil explosion dust, as well as measuring the backscattering echo voltages when laser-detecting targets in the dust without interference from external environmental conditions. Additionally, we analyzed the backscattering echo intensity characteristics of a 905 nm laser at different times and the corresponding positions of the soil explosion dust generated under different masses of explosives, depths of burial (DOBs), and soil moisture contents. We also studied the relationship between the laser backscattering echo intensity and the image gray value of soil explosion dust. This study should be extremely useful for improving the anti-interference ability of laser fuzes and lidars and for realizing the accurate detection and recognition of targets.

## 2. Test Method

### 2.1. Test Platform and Theory

**Figure 1:**
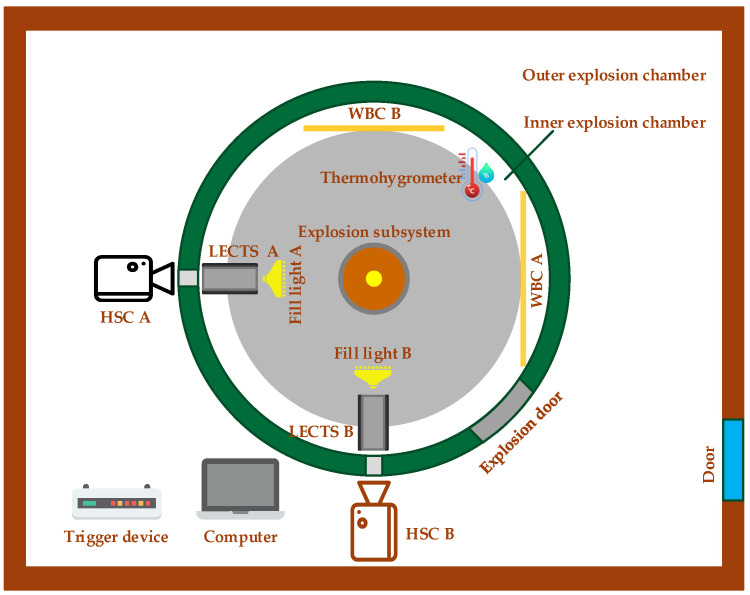
Schematic diagram of the test platform developed for assessing the backscattering echo intensity characteristics of a laser in soil explosion dust. HSC, WBC, and LECTS refer to the high-speed camera, white background cloth, and laser echo characteristic test subsystem, respectively.

**Figure 2:**
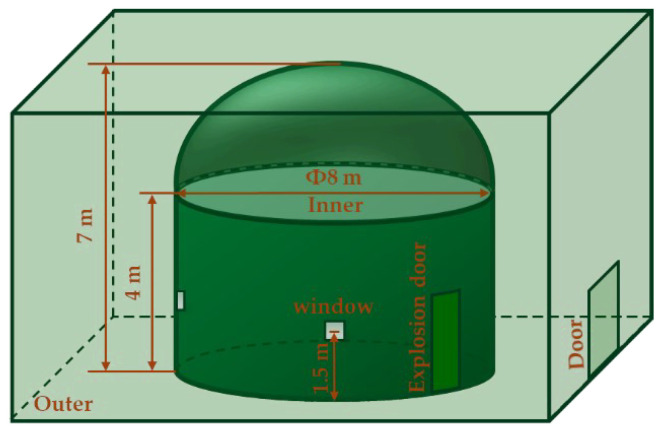
Structure of the explosion chamber.

We used a high-speed camera subsystem to record the formation, diffusion, and setting process of soil explosion dust and used a laser to detect targets and obtain echo voltages in this soil explosion dust. To avoid the influence of external conditions such as temperature, wind speed, and humidity, the test was conducted indoors in an explosion chamber. The test platform (Figure 1) included an explosion chamber, a thermohygrometer, a high-speed camera subsystem, two laser echo characteristic test subsystems, an explosion subsystem, and a trigger device. The explosion chamber (Figure 2) had an inner and outer double-layer structure. The cylindrical side wall of the inner explosion chamber had multiple transparent windows made of explosion-proof glass that acted as windows for testers, high-speed cameras, and other equipment placed in the outer explosion chamber. All personnel were required to evacuate to the outer explosion chamber, and the steel explosion door was closed before the explosive was ignited. This double-layer structure ensured the safety of the test personnel and equipment. A thermohygrometer was used to monitor the temperature and humidity of the inner explosion chamber; the environmental conditions in the inner explosion chamber were kept constant during reduplicative and comparative testing to avoid changes in temperature and humidity that might influence the backscattering echo intensity characteristics of the laser.

**Figure 3:**
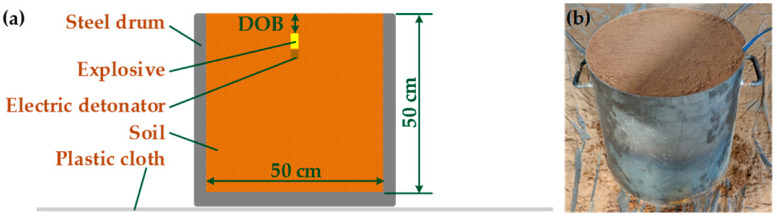
Explosion subsystem: (**a**) internal geometry; (**b**) photograph.

The explosion subsystem (Figure 3) generated different amounts of soil explosion dust, depending on the specific mass of explosive, DOB, and soil moisture content. It contained a steel drum with internal dimensions of Φ 50 × 50 cm and wall thickness of 3 mm. Plastic cloth was placed underneath the steel drum to collect the soil particles scattered by the explosion. The explosive was detonated using an electric detonator. The burial methods for the explosive and electric detonator were those described in [29].

The high-speed camera subsystem comprised a monochrome high-speed camera A, a color high-speed camera B, a 4 × 3 m white background cloth A, a 3 × 2.4 m white background cloth B, two fill lights, and a computer. The two high-speed cameras were vertically distributed to record the formation, diffusion, and settling of the soil explosion dust. To show the size of the soil explosion dust, the white background cloths A and B were marked with horizontal and vertical scale lines at 25 cm intervals along their respective vertical and intersecting edges. They were the targets of laser detection and provided a single background for each high-speed camera, which was convenient for the subsequent image processing and analysis of the soil explosion dust. The fill lights were the light sources for the high-speed cameras, and the computer was used to set the working mode and recording parameters of the high-speed cameras.

The laser echo characteristic test subsystem collected and stored the echo voltages when the laser detected the white background cloth in soil explosion dust. This subsystem emitted a laser beam with a specific repetition frequency and pulse width. After the attenuation of soil explosion dust or targets, the received laser was converted into electrical signals, processed, and stored as voltages. The trigger mode, trigger time, and turn-off delay time of the laser were controlled using an intelligent switch; this allowed the collection of echo data in a particular period, effectively shortening the time for reading the data.

**Figure 4:**
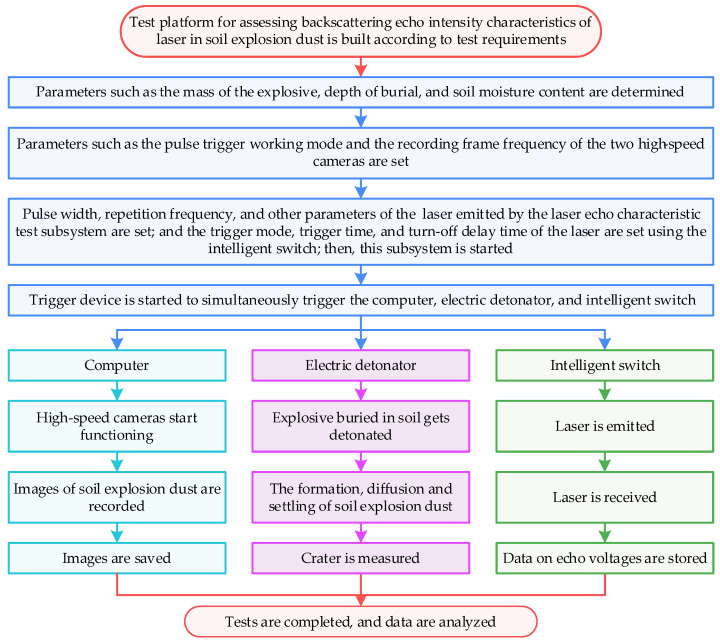
Flowchart of the proposed test method for assessing the backscattering echo intensity characteristics of a laser in soil explosion dust.

The trigger device was connected with the electric detonator, computer, and intelligent switch, simultaneously triggering the explosion subsystem, high-speed camera subsystem, and laser echo characteristic test subsystem, and maintaining the consistency of their time sequences. The flowchart for the test method is shown in Figure 4.

### 2.2. Soil Preparation

**Figure 5:**
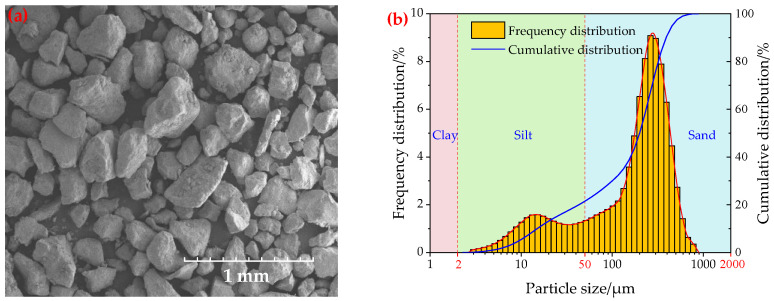
(**a**) Scanning electron microscope image of loamy sand. (**b**) Particle size distributions of loamy sand.

**Figure 6:**
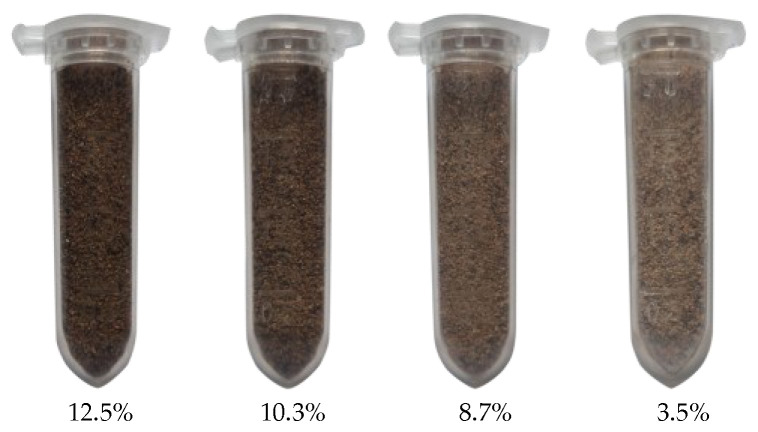
Four kinds of loamy sand differing only in soil moisture content.

The tests were conducted using loamy sand, as defined in the United States Department of Agriculture (USDA, Washington, DC, USA) soil textural triangle [30]; this soil is widely distributed in China. Figure 5a shows the surface morphology of this loamy sand, examined using a SU5000 scanning electron microscope (Hitachi, Japan). The particle size distribution of this loamy sand was assessed using a Horiba LA-950V2 Laser Scattering Particle Size Distribution Analyzer with a measuring particle size range of 10 nm~3 mm (Figure 5b). It is generally believed that the physical properties, such as bulk density, particle size distribution, and soil moisture content, of the soil surrounding a buried explosive have a great impact on crater shape and size, dust volume, and dust diffusion. For this reason, we prepared four kinds of loamy sand that differed only in moisture content: 12.5%, 10.3%, 8.7%, and 3.5% (Figure 6).

### 2.3. Test Plan

**Figure 7:**
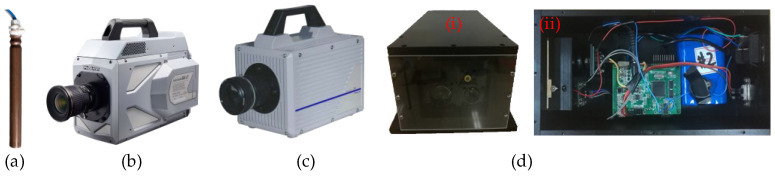
(**a**) #8 electric detonator. (**b**) FASTCAM SA-Z monochrome high-speed camera A. (**c**) FASTCAM SA4 color high-speed camera B. (**d**) Laser echo characteristic test subsystem: (**i**) photograph; (**ii**) internal components.

Nine test series were conducted at the State Key Laboratory of Explosion Science and Technology in the Beijing Institute of Technology, Beijing, China. All the test equipment was laid out according to the schematic diagram of the test platform shown in Figure 1. The cylindrical explosives were made of TNT with masses of 2, 1, and 0.58 g. These explosives were detonated with a #8 electric detonator (Figure 7a). FASTCAM SA-Z (Figure 7b) monochrome and FASTCAM SA4 (Figure 7c) color high-speed cameras (Photron, USA) were used in this study. The lens axes of the monochrome high-speed camera A and color high-speed camera B were perpendicular to the white background cloths A and B, respectively, and passed through the axis of the explosion subsystem. The lenses of these high-speed cameras were 3.8 m from the axis of the explosion subsystem, 6 m from the white background cloth, and 1.6 m from the ground. The laser echo characteristic test subsystems A and B were both equipped with 905D1S 905 nm pulsed laser diodes (Laser Components, Germany), which could emit laser beams with a pulse repetition frequency of 1 Hz~10 kHz, pulse width of 20~100 ns, and sampling frequency of 250 MHz (Figure 7d).

The test plan comprised four parametric studies, each of which contained several individual test series. The details of the nine test series that were conducted are summarized in Table 1.

(1) Influence of mass of explosive: Series A~C

A: The recording frame rates of FASTCAM SA-Z monochrome and FASTCAM SA4 color high-speed cameras were 5000 and 500 fps, respectively. The axis of the laser beam of the laser echo characteristic test subsystem A was perpendicular to the white background cloth A and passed through the axis of the explosion subsystem. The laser was 3.3 m from the axis of the explosion subsystem, 5.5 m from the white background cloth, and 70 cm from the top surface of the explosion subsystem. The mass of the explosive *m* was 2 g. The laser echo characteristic test subsystem B was not used.

B: Same setup as A, except that *m* = 1 g.

C: Same setup as A, except that *m* = 0.58 g.

(2) Influence of DOB: Series D~F

D: Same setup as B, except that the laser was 65 cm from the top surface of the explosion subsystem. The laser echo characteristic test subsystems B and A were set up in the same way, except that the height to the top surface of the explosion subsystem was 80 cm. The DOB of the explosive was 3 cm.

E: Same setup as D, except that DOB = 0 cm.

F: Same setup as D, except that DOB = 8 cm.

(3) Influence of soil moisture content: Series D, G~I

D: Soil moisture content *w* was 12.5%.

G: Same setup as D, except that *w* = 10.3%.

H: Same setup as D, except that *w* = 8.7%.

I: Same setup as D, except that *w* = 3.5%.

(4) Influence of laser irradiation position: Series B & D

B: The height of the axis of the laser beam emitted by the laser echo characteristic test subsystem A above the top surface of the explosion subsystem was 70 cm.

D: The heights of the axes of the laser beams emitted by the laser echo characteristic test subsystems A and B above the top surface of the explosion subsystem were 65 and 80 cm, respectively.

## 3. Results and Discussion

### 3.1. Crater

**Figure 8:**
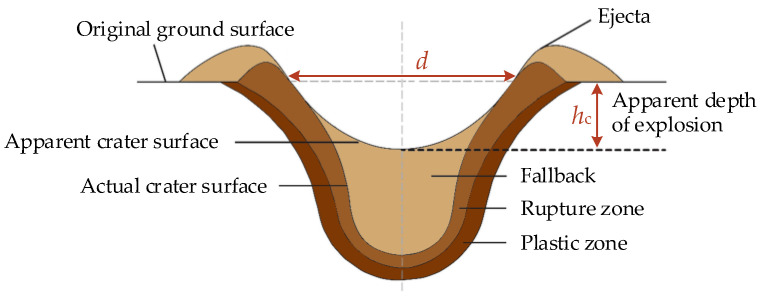
Schematic diagram of crater.

An explosion causes the displacement and ejection of soil from the ground; if the explosion is close to the surface, a crater is formed by the complex interaction of gravity, soil strength, and transient load conditions [31]. As shown in Figure 8, a crater is divided into two categories: the apparent crater and the actual crater [32]. The apparent crater is visible on the surface. The actual crater is filled with loose and almost discrete materials from the explosion. In the rupture zone, the materials below the actual crater are crushed and cracked but have no significant displacements. In the plastic zone, particles exhibit slight permanent displacements that are very small in the vicinity of the elastic zone.

**Figure 9:**
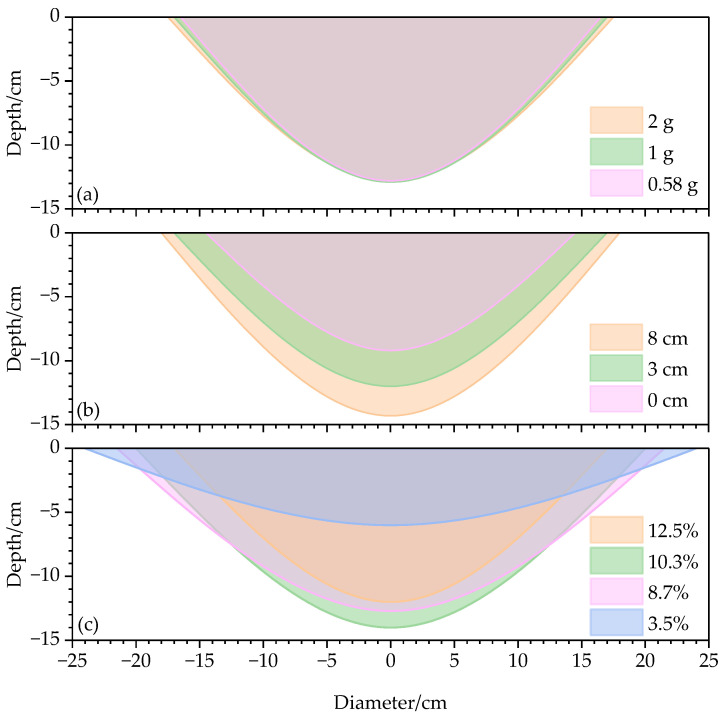
Sizes of apparent craters under different (**a**) masses of explosive, (**b**) depths of burial (DOBs), and (**c**) soil moisture contents.

The mass of the explosive, DOB, and soil moisture content are the most critical parameters affecting the shape and size of the crater. The diameter *d* of the craters in our study increased with the increasing mass of the explosive (Figure 9a). However, the apparent depth did not significantly change whether the mass of the explosive was 2, 1, or 0.58 g. When the DOB increased, larger amounts of subsoil were expelled by the explosion, leading to an increase in the crater diameter and apparent depth (Figure 9b). There was a significant negative correlation between the soil moisture content and crater diameter, while the apparent depth first increased and then decreased with decreasing soil moisture content (Figure 9c).

**Figure 10:**
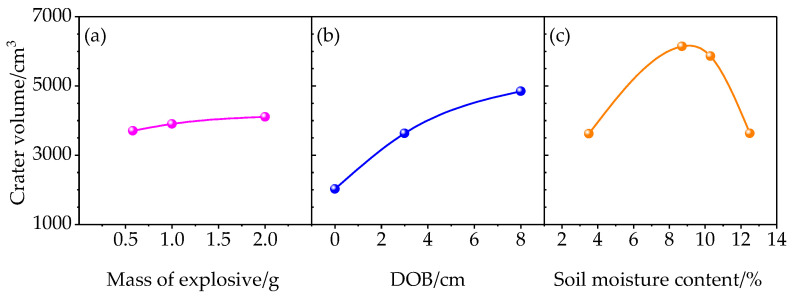
Crater volumes under different (**a**) masses of explosive, (**b**) DOBs, and (**c**) soil moisture contents.

Assessing craters is an appropriate tool for studying blast phenomena and the destructive power of different explosives. The crater volume is closely related to the mass of soil explosion dust [31]. If the crater is approximated as a cone, its volume, *V*, is given as [33]:

(1) $$V=\pi d^{2}h_{c}/12,$$

where *d* is the actual crater diameter and *h*_c_ is the apparent depth of the explosion. The crater volume *V* was positively correlated with the mass of the explosive and DOB; it increased with increasing soil moisture content until a certain threshold value, above which it decreased (Figure 10).

The above results showed that as the DOB and the mass of the explosive increased, more soil was moved by the explosion. As the soil moisture content was reduced, the soil cohesion among the particles became smaller, and the soil resistance decreased. Under the influence of the generated shock wave, more soil around the explosive was thrown out, increasing the apparent depth. However, when the soil moisture content fell below a certain threshold, the soil cohesion was no longer enough to drive the movement of nearby particles. When the explosion occurred, only the soil around and under the explosive was thrown out. Meanwhile, the soil around the explosion crater collapsed, and the lower part of the blasting crater was buried because of the detonation. Therefore, the apparent depth became smaller. Similarly, the surface layer over the soil on the top of the explosive was air, and the air resistance on the soil was minimal. When the soil cohesion between the soil particles was reduced, the soil on the top of the explosive was more likely to be thrown out by the shock wave; thus, the diameter of the crater became larger. Correspondingly, the crater volume first increased and then decreased with the decrease in the soil moisture content.

### 3.2. Temporal and Spatial Distribution of Soil Explosion Dust

The physical process that occurs following the detonation of an explosive buried in soil has four phases:

(1) Phase 1: explosion and early interaction with the soil;
(2) Phase 2: gas expansion;
(3) Phase 3: generation of smoke and soil ejecta;
(4) Phase 4: diffusion and settling of soil explosion dust.

Under the action of detonation, the first two stages occur very rapidly (in ms). From phase 3, the soil explosion dust forms and diffuses; then, it is fully dispersed and finally, settles to the ground under the influence of gravity. In phase 3, it may contain considerable smoke; however, it is composed mostly of soil particles in phase 4.

**Figure 11:**
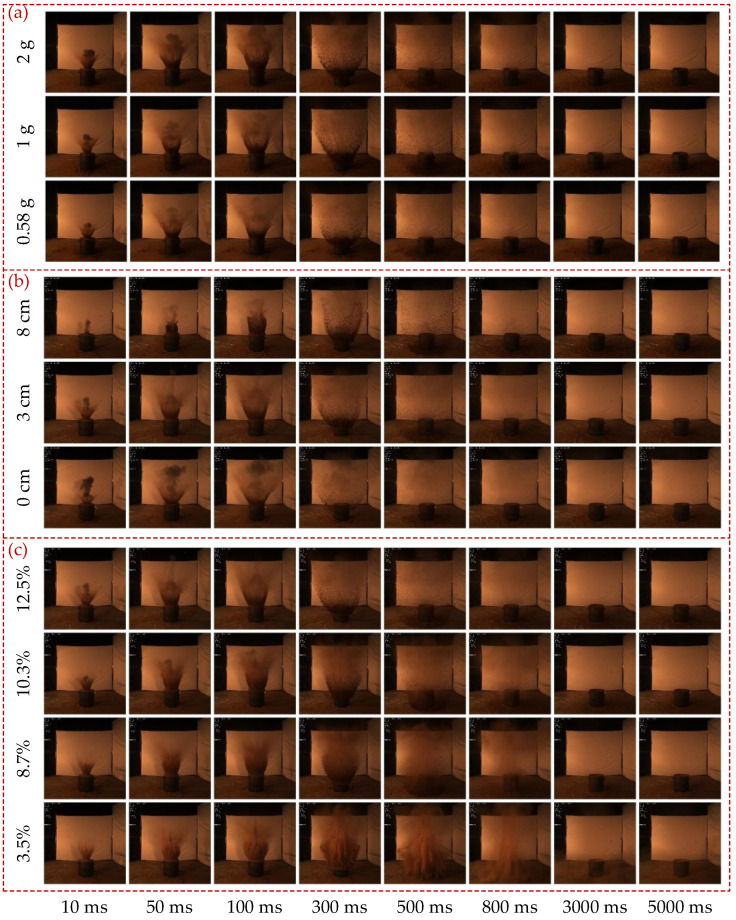
Temporal and spatial distribution characteristics of soil explosion dust under different (**a**) masses of explosive, (**b**) DOBs, and (**c**) soil moisture contents.

The diffusion speed of soil explosion dust was very fast in the first 3 s, and the concentration of dust was the highest in the first 500 ms (Figure 11). Most soil explosion dust settled within 5 s, but some tiny particles still floated in the air. The distribution of the soil explosion dust was approximately symmetrical around the axis of the explosion subsystem.

The smoke produced by the explosion mainly appeared in phase 3 and had almost disappeared before 100 ms (Figure 11). The concentration and diffusion domain of the visible smoke increased with the mass of the explosive but decreased with DOB. This was not only because larger masses of explosive generated more smoke but also because more visible smoke was released into the air when the DOB was shallower. Additionally, when the soil was dry enough, less smoke was produced by the explosion, and the soil explosion dust was more diffuse and had a stronger shielding effect on the smoke. Therefore, the smoke was barely visible when the soil moisture content was 8.7% or 3.5%.

In phase 4, the soil explosion dust first spread upwards and outwards under the action of the shock wave, then fell back under the action of gravity. The concentration of soil explosion dust decreased from bottom to top, and its mass was positively correlated with the crater volume (Figure 11). For the diffusion phase of soil explosion dust in phase 4, at a given time, the concentration of soil explosion dust was positively correlated with the mass of the explosive; increasing this mass caused the explosion to generate more energy to drive more soil to spread out (Figure 11a). Furthermore, within a certain range of DOB, shallower DOB implied that less soil was ejected and more energy was imparted to each dust particle, resulting in a smaller dust concentration and larger diffusion domain (Figure 11b). Additionally, as the crater volume first increased and then decreased with increasing soil moisture content, the diffusion speed of soil explosion dust first decreased and then increased under the same detonation energy. Therefore, the diffusion domain first decreased and then increased with the increasing soil moisture content, and the concentration of soil explosion dust did the opposite (Figure 11c). However, the time required for the diffusion and settling of soil explosion dust was inversely proportional to soil moisture content. In short, the distribution characteristics of the soil explosion dust in the formation and early diffusion phase are similar to those in [24]; the variation of dust concentration in the diffusion phase is similar to that of crater volume.

### 3.3. Laser Backscattering Echo Intensity Characteristics

To study the backscattering echo intensity characteristics of lasers in soil explosion dust, we measured the echo voltages as the 905 nm laser detected the target of white background cloth in varying dust concentration fields. To some extent, this measurement method is similar to the approach described in [34]. Since the backscattering echo intensity from the white background cloth was greater than that from the soil explosion dust, the peak echo voltage of the laser was the largest at the beginning of the detonation and was inversely related to the dust concentration. The normalized peak echo voltage, the ratio of the backscattering echo voltage with interference from soil explosion dust to that without such interference, was used to analyze the influence of the mass of the explosive, DOB, soil moisture content, and laser irradiation position on the backscattering echo intensity characteristics. Smaller normalized peak echo voltages indicated more significant interference effects from the dust.

**Figure 12:**
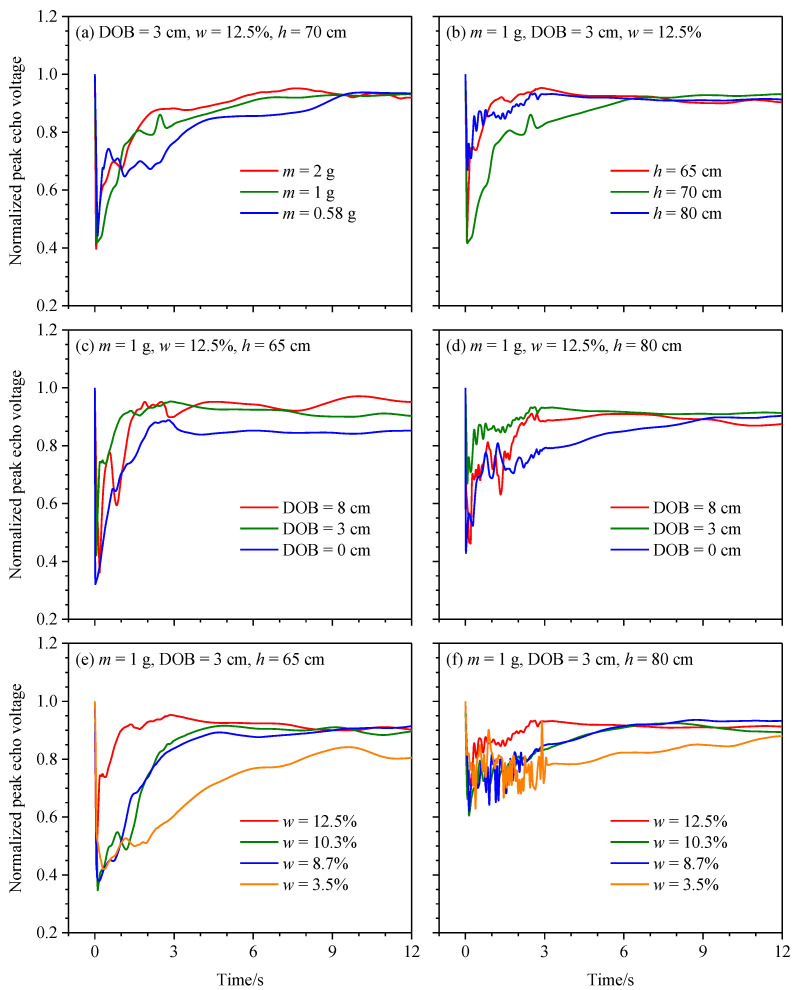
Normalized peak echo voltages under different masses of explosive, DOBs, soil moisture contents, and heights of the laser irradiation positions.

The height *h* of the laser irradiation position is the height of the axis of the laser beam emitted by the laser echo characteristic test subsystem A or B above the top surface of the explosion subsystem. The peak echo voltage at the laser irradiation position changed sharply in the first 3 s (Figure 12). In the early phase of the detonation, the concentration of soil explosion dust surged to the maximum value, and the interference effect on the laser was strongest: the peak echo voltage decreased sharply from its maximum value of 1 to its minimum value. In the diffusion phase, the dust concentration decreased in fluctuations at the same position, leading to an oscillating increase in the peak echo voltage. In the later phase of dust diffusion, the dust was fully dispersed, and the increasing rate of the peak echo voltage was gradually reduced. However, in the early phase of dust settling under the action of gravity, the concentration at the laser detection position increased slightly, then decreased gradually, and finally, arrived at a stable value. Correspondingly, the peak echo voltage first decreased slightly and then increased, finally stabilizing and approaching a value of 1. For the same mass of explosive, DOB, and soil moisture content, higher laser irradiation positions corresponded to greater fluctuations in the dust concentration; correspondingly, the oscillation in the peak echo voltage of the laser became more pronounced. The minimum normalized peak echo voltage ranged from 0.318 to 0.658 under different test conditions (Figure 12), indicating that the interference effect of soil explosion dust on the laser cannot be ignored. Assuming that the laser can accurately detect the white background cloth target in this soil explosion dust environment, the normalized peak echo voltage corresponding to the target determination threshold of the laser should be less than the minimum normalized peak echo voltage. Hence, it is essential to further analyze the relationships among the minimum normalized peak echo voltage, mass of explosive, DOB, soil moisture content, and height of the laser irradiation position.

**Figure 13:**
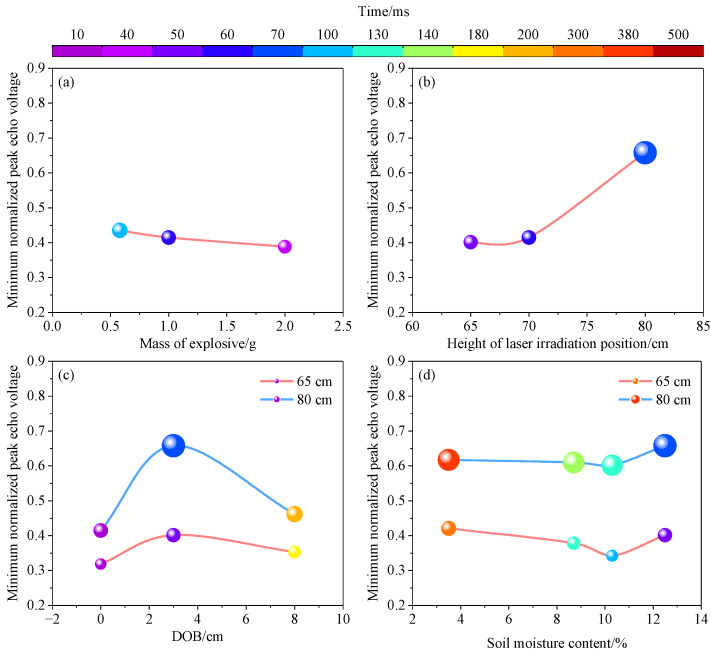
Minimum normalized peak echo voltages and times to reach the minimum under different (**a**) masses of explosives, (**b**) heights of laser irradiation positions, (**c**) DOBs, and (**d**) soil moisture contents.

Figure 13 shows that the time required to reach the minimum normalized peak echo voltage is less than 500 ms, and it is inversely related to the mass of the explosive and the soil moisture content, but positively proportional to the height of the laser irradiation position and the DOB of the explosive. The minimum normalized peak echo voltage was inversely related to the mass of the explosive but positively related to the height of the laser irradiation position. The minimum normalized peak echo voltage first decreased and then increased with the increase in the soil moisture content, while the DOB showed the opposite behavior. The large quantity of visible smoke produced by the explosion when the DOB was close to zero significantly affected the laser, resulting in a smaller minimum normalized peak echo voltage.

### 3.4. Relationship between Echo Intensity and Image Gray Value

The above analysis revealed that the variation law of the peak echo voltage of the laser was similar to that of the image of the soil explosion dust. We used the gray value of the monochrome image of soil explosion dust to characterize its concentration, extracted the mean gray value of the monochrome image (with 50 px corresponding to the laser irradiation position), and then compared the mean gray value at each moment with that at the initial moment (when only the white background cloth was captured) to obtain a normalized mean gray value. We carried out a Spearman rank correlation analysis between the normalized mean gray value of the monochrome image and the normalized peak echo voltage of the laser. The Spearman rank correlation describes the monotonic relationship between two variables. It is useful for nonnormally distributed continuous data and ordinal data and is relatively robust to outliers [35]. The Spearman rank correlation coefficient (SRCC) $r_{s}$ can be given as [36]:

(2) $$r_{s}=\frac{\sum_{i=1}^{n}\left(x_{i}-\bar{x}\right)\left(y_{i}-\bar{y}\right)}{\sqrt{\sum_{i=1}^{n}{\left(x_{i}-\bar{x}\right)}^{2}\sum_{i=1}^{n}{\left(y_{i}-\bar{y}\right)}^{2}}},$$

where $x_{i}$ and $y_{i}$ are the ranks of the *i*th data of the random variables *X* and *Y*, respectively.

**Figure 14:**
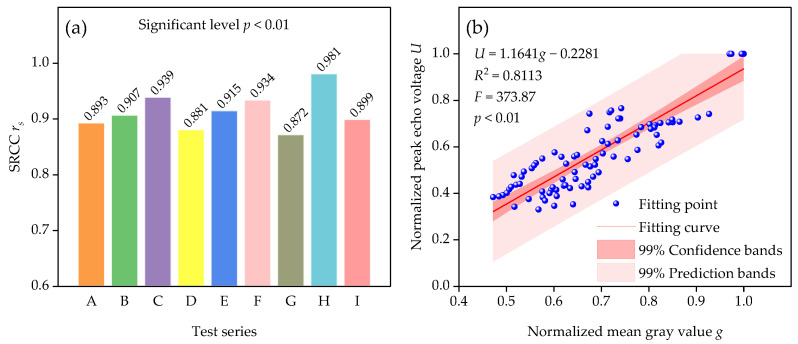
(**a**) SRCC values of test series A~I. (**b**) Correlation between normalized peak echo voltage and normalized mean gray value.

We calculated the SRCC values of the nine test series A~I (Figure 14a). We found that the normalized peak echo voltage *U* of the laser had a very strong positive correlation with the normalized mean gray value *g* of the monochrome image of soil explosion dust at the significant level 0.01. Further, ten pairs of equally time-spaced data were selected from each test series A~I to obtain 90 pairs of data to estimate the relationship between *U* and *g* by fitting the model equations via linear regression with 99% confidence intervals. Figure 14b shows that *F* = 373.87 and *p* < 0.01. Therefore, the null hypothesis that the regression coefficient is 0 is rejected at level 0.01, and the model equation *U* = 1.1641*g* − 0.2281 meets the requirements. In addition, the regression coefficient was significant at level 0.01. The coefficient of determination *R*^2^ suggested that about 81.13% of the variability of the normalized peak echo voltage of the laser could be explained by the relationship with the normalized mean gray value of the monochrome image of soil explosion dust.

## 4. Conclusions

In the present study, we propose a test method utilizing high-speed cameras and an explosion chamber to assess the backscattering echo intensity of lasers in dust generated by small-scale explosive blasts in soil. With this approach, we measured the backscattering echo voltages of a 905 nm laser while detecting a white background cloth in soil explosion dust. The conducted tests considered the influence of the mass of explosive (0.58 g, 1 g, 2 g), DOB (0 cm, 3 cm, 8 cm), soil moisture content (3.5%, 8.7%, 10.3%, 12.5%), and height of laser irradiation position (65 cm, 70 cm, 80 cm). The main conclusions are summarized as follows. The concentration of soil explosion dust was found to be highest in the first 500 ms; most soil explosion dust settled within 5 s. Moreover, the backscattering echo intensity was inversely proportional to the dust concentration, and the minimum normalized peak echo voltage ranged from 0.318 to 0.658, indicating that soil explosion dust interfered significantly with detecting the white background cloth target. Furthermore, the minimum normalized peak echo voltage was inversely related to the mass of the explosive but positively related to the height of the laser irradiation position, and it first decreased and then increased with the increase in the soil moisture content; DOB had the opposite effect from soil moisture content. Additionally, the mean gray value of the monochrome image of soil explosion dust had an extremely strong positive correlation with the backscattering echo intensity of the laser; it can therefore be used to study the laser’s detection performance.

This test method has the strengths of simultaneously recording the temporal and spatial distribution of soil explosion dust and measuring the backscattering echo voltages of the laser without interference from the external environment such as temperature, humidity, and wind speed. However, the explosion chamber only allows the detonation of small-scale explosives, so this approach is not suitable for the test involving an explosive with a mass exceeding 100 g. This work provides experimental data for verifying simulations of the backscatter echo intensity of lasers in soil explosion dust and setting reasonable thresholds for laser detection and target recognition. It is thus of great significance for improving the detection accuracy and anti-interference ability of lasers. However, due to constraints such as limited resources and experimental costs, only nine test series were conducted in our study; we will adopt a combined approach of simulation and experimentation to comprehensively assess the backscattering echo intensity characteristics of lasers in soil explosion dust in the future.

## Figures and Tables

**Table 1:** Summary of the experimental test plan.

Series	Soil Type		Explosive	
	Moisture Content *w*/%	Bulk Density *ρ*/(g/cm^3^)	Mass *m*/g	DOB/cm
A	12.5	1.80	2	3
B	12.5	1.80	1	3
C	12.5	1.80	0.58	3
D	12.5	1.80	1	3
E	12.5	1.80	1	0
F	12.5	1.80	1	8
G	10.3	1.76	1	3
H	8.7	1.74	1	3
I	3.5	1.66	1	3

## Data Availability

Data underlying the results presented in this paper are not publicly available at this time but may be obtained from the authors upon reasonable request.
